# The neuroanatomical organization of the hypothalamus is driven by spatial and topological efficiency

**DOI:** 10.3389/fnsys.2024.1417346

**Published:** 2024-08-05

**Authors:** Nathan R. Smith, Shabeeb Ameen, Sierra N. Miller, James M. Kasper, Jennifer M. Schwarz, Jonathan D. Hommel, Ahmad Borzou

**Affiliations:** ^1^Center for Addiction Sciences and Therapeutics, Department of Pharmacology and Toxicology, University of Texas Medical Branch, Galveston, TX, United States; ^2^Physics Department and BioInspired Institute, Syracuse University, Syracuse, NY, United States; ^3^Indian Creek Farm, Ithaca, NY, United States; ^4^CompuFlair, Houston, TX, United States

**Keywords:** Allen Brain Atlas, computational biology, connectome, connectivity, efficiency, hypothalamus, graph theory - graph algorithms, Monte - Carlo simulation

## Abstract

The hypothalamus in the mammalian brain is responsible for regulating functions associated with survival and reproduction representing a complex set of highly interconnected, yet anatomically and functionally distinct, sub-regions. It remains unclear what factors drive the spatial organization of sub-regions within the hypothalamus. One potential factor may be structural connectivity of the network that promotes efficient function with well-connected sub-regions placed closer together geometrically, i.e., the strongest axonal signal transferred through the shortest geometrical distance. To empirically test for such efficiency, we use hypothalamic data derived from the Allen Mouse Brain Connectivity Atlas, which provides a structural connectivity map of mouse brain regions derived from a series of viral tracing experiments. Using both cost function minimization and comparison with a weighted, sphere-packing ensemble, we demonstrate that the sum of the distances between hypothalamic sub-regions are not close to the minimum possible distance, consistent with prior whole brain studies. However, if such distances are weighted by the inverse of the magnitude of the connectivity, their sum is among the lowest possible values. Specifically, the hypothalamus appears within the top 94th percentile of neural efficiencies of randomly packed configurations and within one standard deviation of the median efficiency when packings are optimized for maximal neural efficiency. Our results, therefore, indicate that a combination of geometrical and topological constraints help govern the structure of the hypothalamus.

## Introduction

The mammalian brain is a complex network composed of functionally and anatomically distinct regions. As our ability to identify regions in the brain with distinct cell types and molecular markers increases, our understanding of how different brain regions are organized spatially becomes vital if we hope to understand the impact of these regions in physiology and pathology as structure can be strongly coupled to functionality (Maynard et al., 2021). Furthermore, the brain’s functionality appears to depend largely on the neuronal distribution between brain regions, commonly referred to as the connectome. The connectome can be represented as a graph, where nodes represent brain regions and edges represent axonal projections between regions. Such a graph for the whole brain displays some universal characteristics such as modularity (Meunier et al., 2010) and small-worldness (Watts and Strogatz, 1998; Bassett and Bullmore, 2006) which may have emerged to improve the communication quality of the neural network (Bullmore and Sporns, 2012; Mišić et al., 2015). Such graphs exist independent of spatial orientation and physical packing (i.e., spatial embedding) of the individual nodes within the brain. However, spatial embedding is an important aspect underlying the neuronal connectome.

As brain regions are laid out in a manner constrained by their physical volume in three-dimensional space, spatial embedding may be driven by the advantages of an efficiently organized network – measures that involve a combination of geometry and network topology. To be precise, one possible driving factor may be the minimization of the “wiring cost” defined as the total length of axonal projections between brain regions measuring the minimality in material usage of the neuronal network (Kaiser and Hilgetag, 2006; Bassett et al., 2010). Rubinov et al. explored the brain’s organization in terms of wiring cost and found that, if wiring cost is defined as either axonal distance between regions or the product of axonal distance and axonal bandwidth, the mouse connectome cannot be explained entirely by the global minimization of wiring cost (Rubinov et al., 2015). Given this finding, they argued that there is a trade-off between wiring cost and high-participation hubs to enhance connectome-mediated communication between functionally distinct regions. The efficiency of such high-participation hubs (or connector hubs) are potentially measured directly by the small-world efficiency index (Latora and Marchiori, 2001). Looking at 55 areas of the cat cortex, it was found to be 69% small-world efficient as compared with 57% small world efficiency in a random graph. However, more recent analysis suggests only weak small-world properties with some networks in the brain (Swanson et al., 2019), indicating the need for a more refined quantitative strategy to measure efficiency. As the brain’s spatial structure and wiring together drive its organization, an efficiency measure that simultaneously takes these factors into account is of interest. On the one hand, the wiring cost does not take the magnitude of the axonal projections (edges) by the connectome. On the other hand, the small-worldness does not take the spatial distances into account. Therefore, we propose a complementary measure defined as “neural efficiency” which is maximized when the axonal projections of the highest magnitude are sent through the shortest possible neuronal paths.

Here, we focus on the organizational efficiencies of the hypothalamus. The hypothalamus plays a key role in activities that are essential for the survival of the body (Swanson, 1986; Swanson, 1987; Swanson, 2000; Simerly, 2015). It maintains the body’s homeostasis by controlling factors such as temperature, hunger and satiety, and cardiovascular regulation. In addition to its vitality, the hypothalamus’ functionality is diverse (Swanson, 2000; Simerly, 2015), which can be due to its topological and wiring complexities. This brain’s sub-region holds the densest wiring of the whole central nervous system (Hahn and Swanson, 2010; Hahn and Swanson, 2012; Hahn and Swanson, 2015). Despite the important functionality of the hypothalamus, there has not been much focus on the organization of the hypothalamic neuronal network (Bedont et al., 2015; Hahn et al., 2019). Recent network analysis for the human hypothalamus demonstrates that that there are two interconnected sub-networks each with their own sub-structures with possible implications for future hypothesis-driven work (Hahn et al., 2019). Here, we take a step back from such detailed, hypothalamic network analysis to look for more minimal principles of structural organization. Specifically, we draw data from a database of viral tracing experiments to construct a model representation of the wild-type mouse hypothalamus that reflects its (i) neural connectivity (network configuration), (ii) magnitude of the axonal projections (edges), and (iii) spatial position of hypothalamic subregions (nodes). We demonstrate that neither the unweighted small-world efficiency nor the wiring cost of the network is in its highest optimal state in the mouse hypothalamus. However, our new definition of neural efficiency, which accounts for all three aforementioned characteristics together, shows that the hypothalamic network is indeed organized efficiently. This new understanding demonstrates the anatomical properties which underlie the hypothalamic network and suggests new characteristics to study in the context of both neurological diseases and artificial networks.

## Methods

The Allen Mouse Brain Connectivity Atlas provides a structural connectivity map of the mouse brain regions derived from a series of 499 viral tracing experiments comprising 157 brain regions in the wild-type mouse (Oh et al., 2014). We employed this connectivity data coupled with positional data also taken from the Atlas for 26 individual brain sub-regions of the hypothalamus in the wild-type model.

For each brain region listed in the Allen Brain Connectivity Atlas, the Atlas gives the region’s volume, injection position, and axonal projection volumes to target structures measured using viral tracing experiments. We utilized these data to generate a simple model of the complex mouse brain. We limited our analysis to brain regions that were the targets of injections, which would therefore have mapped axons arising from them. We assumed that, while axons do arise from these excluded regions, they are likely insufficiently characterized by the Atlas for our analysis. For each brain region, the Atlas carries duplicate viral injection experiments. We arbitrarily chose to sample the experimental projections with the greatest intensity for each brain region since our preliminary analysis showed the injection experiment chosen did not significantly impact results. Further, the authors of the Atlas found that the projection intensities of sets of duplicate injections differed only within one standard deviation (Oh et al., 2014). Additionally, while brain region locations are well-mapped in the mouse brain, injection positions differ slightly between experiments. To define the experimental 3D position of a given brain region, we took the geometric average of all injection positions from experiments for the region after preliminary analysis using different, specific experiment injection coordinates yielded similar data.

The Allen Brain Connectivity Atlas utilizes quality control steps to ensure inclusion of only axonal projection fluorescence in its anterograde projectome data. The dendrites of AAV-infected neurons may contribute to overall projection intensity if not accounted for. To combat this, the Atlas created a polygonal exclusion zone surrounding the injection site to remove dendritic signal intensity for more accurate informatics processing. The inclusion areas were translated to the Allen Mouse Common Coordinate Framework and the areal proportion of each infected structure was obtained (Allen Institute for Brain Science, 2017).

To simplify the brain’s geometry, and thereby reduce the computational complexity, we assumed that each region is approximately spherical and is represented in our model as a sphere of the region’s same volume. In Appendix B, we show whether brain regions are truly spherical bears no impact on the results or validity of our model. Additionally, we only addressed one hemisphere, assuming laterality plays a minimal role in efficiency. With these assumptions in place, we modeled the brain’s layout as a set of spheres in locations approximately true to those derived from their viral injection sites. The cost of this simplification is that 34 out of 325 possible region pairs have undesired, but relatively negligible, overlaps. The average of the overlaps is only 0.2 (mm) and the maximum overlap is 0.8 (mm). In addition to the spatial information, we retrieved the axonal projection volumes and the connectivity map, i.e., the weighted adjacency matrix, of the mouse hypothalamus from the Allen Brain Atlas.

We hypothesize that the hypothalamus is developed such that the highest magnitude of axonal signaling is transmitted between its sub-regions over the shortest distance possible. In other words, the minimization of spatial distances between the sub-regions of hypothalamus competes with the maximization of the magnitude of the axonal signals transmitted between the sub-regions such that neither of the two factors is highly optimal alone. Therefore, we postulate that the following measure of efficiency is maximized in the hypothalamus:

(1) $$S=\underset{ij}{\sum }\frac{I_{ij}}{d_{ij}}$$

Where *i* and *j* enumerate all the possible pairs of the sub-regions, *d_ij_* is the Euclidean distance between the two regions *i* and *j* and $I_{ij}$ is the axonal projection volume between regions *i* and *j*. This value obtained from the Allen Brain Atlas is in units of (mm^3^). Given the asymmetric nature of the $I_{ij}$ matrix, the wiring in the hypothalamus is represented by a directed graph. Inserting the data from the Atlas into Eq. (1) returns an efficiency value of 24.2 (mm^2^). Note that for the densely connected hypothalamus, Euclidean distance is a reasonable metric since the neuronal pathways are not curved.

**Figure 1:**
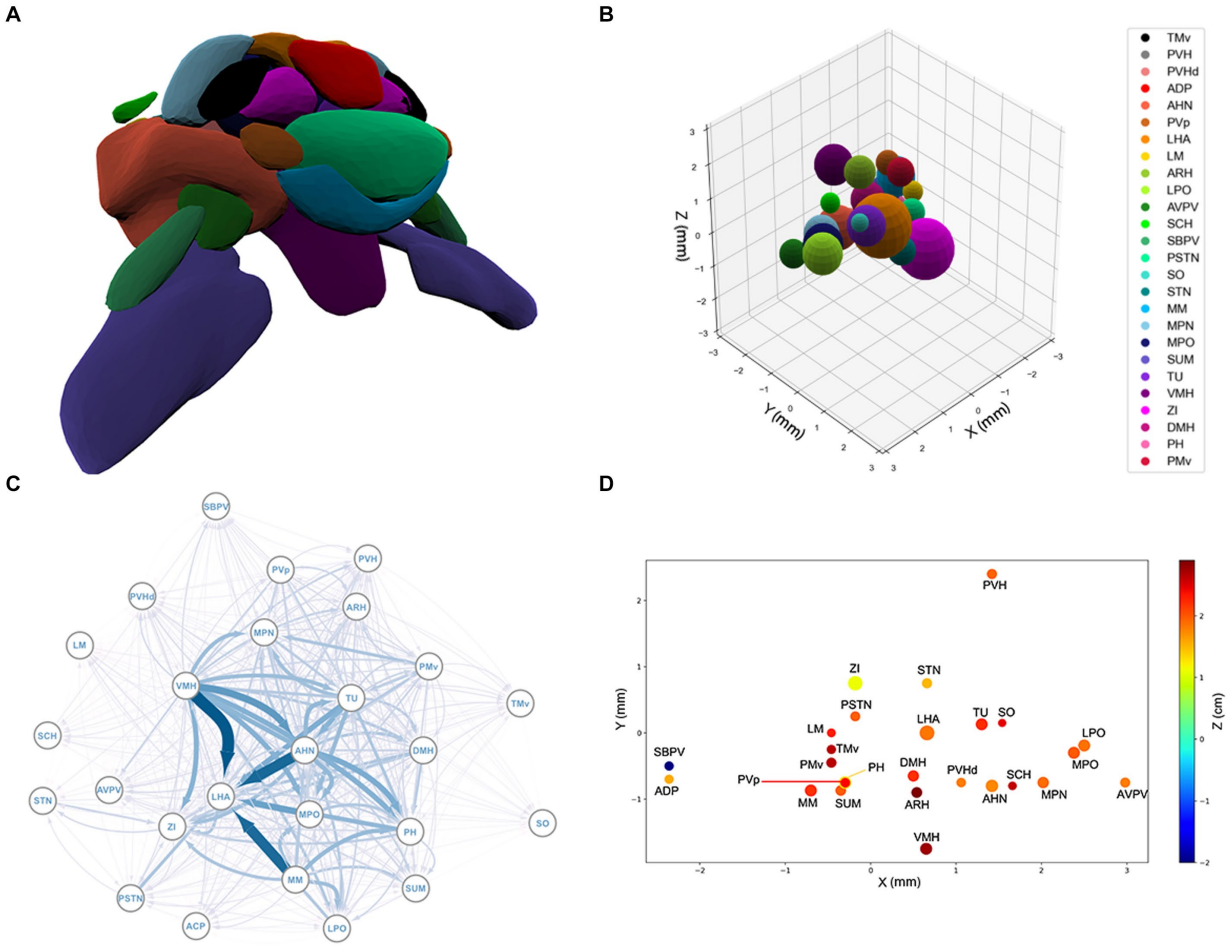
**(A)** The spatial structure of the 26 sub-regions of hypothalamus derived from the Allen Brain Atlas. The outlier regions, PVH, ADP, and SBPV, are removed. **(B)** The spherical model of the sub-regions of the hypothalamus. For a list of sub-region abbreviations, see Appendix A. **(C)** Graphical representation of the hypothalamic network. Each node represents a brain region and each arrow an axonal projection. The width of the arrows represents the axonal volume. **(D)** 3D projection of the spatial structure of the hypothalamus. See Appendix A for a list of abbreviations.

To determine whether the mouse hypothalamus is laid out efficiently according to our definition, we used computational methods to derive a model hypothalamus to maximize efficiency to compare to the Atlas-derived hypothalamus. We kept the wiring configuration, i.e., the weighted adjacency matrix $I_{ij}$ , constant, generated an array of 26 random positions each of the spherical regions, and minimized the inverse of Eq. (1) (thereby maximizing the efficiency, *S*), using the Minimize method of the Optimize class in the SciPy library for the Python programming language. The optimizer starts with the random packing and explores the 26×3 dimensional phase-space to find a set of (x, y, z) positions for each of the 26 regions that maximizes Eq. (1). Since the brain regions occupy a certain volume in the physical space, the minimization is under the constraint that only a negligible overlap, equivalent or less than the overlaps of the spheres in Figure 1, is allowed. Among the possible minimization methods that are implemented in SciPy (Virtanen et al., 2020), we use SLSQP (Kraft, 1988), which can accommodate the constraints. The (*x*, *y*, *z*) variables are bound to move between 0 and 15 (mm). We report that the outcome is independent of the perturbations in the upper limit, and independent of upper limits greater than 15 (mm). This process is repeated $10^{4}$ times to obtain an ensemble of hypothalamus packings.

To complement the optimization study, we also conducted an independent random-packing investigation. This study explores the phase-space of possible positions for the hypothalamus sub-regions and quantifies how the efficiency of the true configuration of hypothalamus compares to efficiencies on a relevant region of this phase space. To facilitate the building of a suitable ensemble of possible configurations, we first reduced the phase space dimensions to 23×3 by considering only the sub-network in the bulk of the hypothalamus, removing from the network the three distinct outer regions as seen in Figure 1B.^1^ The volumes of the remaining regions, modeled as spheres, are the same as the true ones in the hypothalamus. Moreover, as in the optimization study, in all the random packings, the adjacency matrix $I_{ij}$ is constant (although, with the three nodes and their connections removed, the relevant elements of $I_{ij}$ now belong to a 23×23 submatrix).

**Figure 2:**
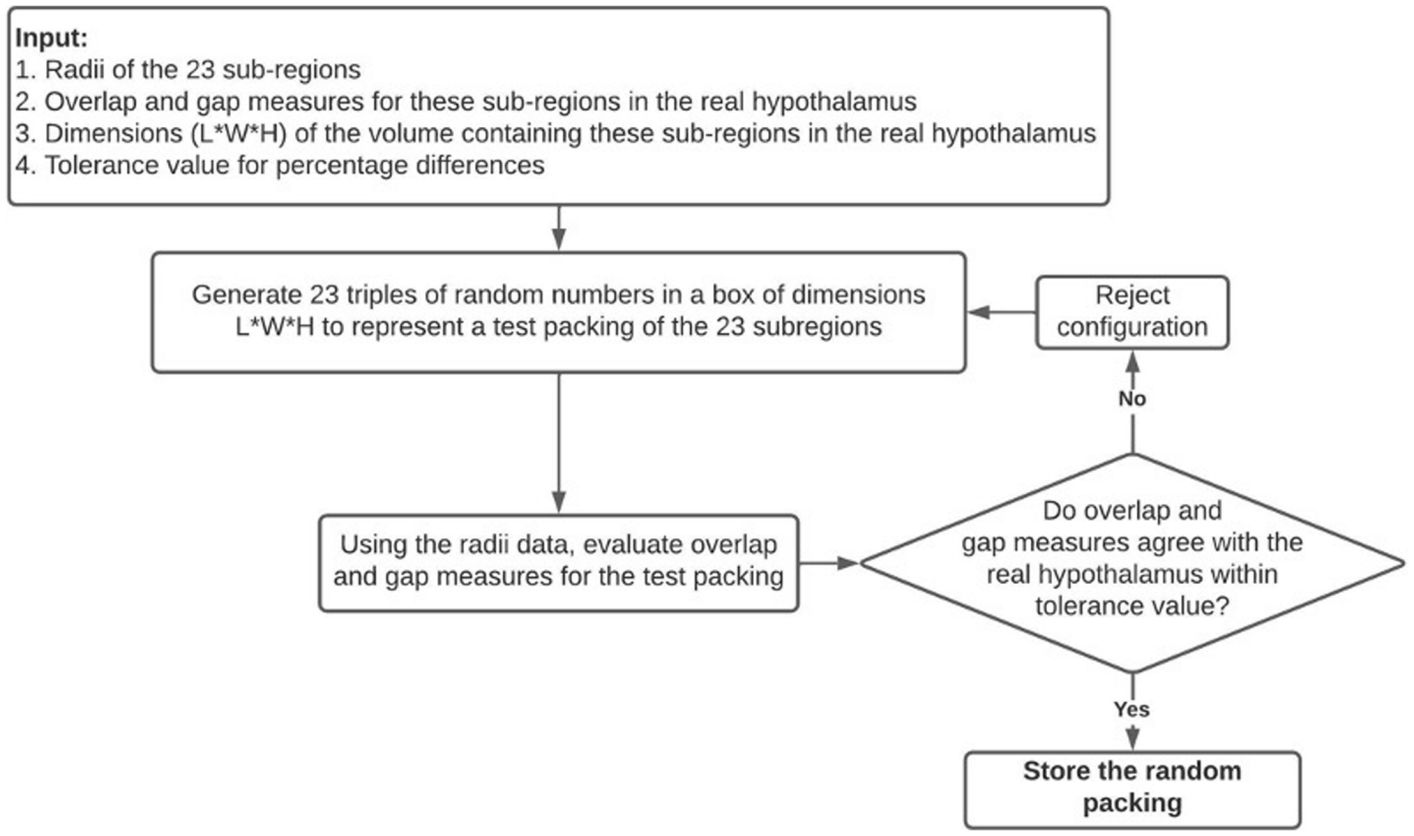
A visual summary of the random packing algorithm used to generate theoretical hypothalamic configurations to compare to the true configuration of the hypothalamus.

Suitable random packings of these regions are generated using the Numpy library’s random number generator and the following algorithm which is summarized in Figure 2:

- A random configuration is first proposed by generating 23 coordinates within a 3D box with dimensions that would barely fit the bulk of the hypothalamus.
- The configuration is compared to the bulk real hypothalamus by evaluating numerical measures of overlaps and gaps. The difference between the center-to-center distance and the sum of radii of two edges is called an overlap if it has a negative value, and a gap if it has a positive value. The following six quantities are evaluated on the proposed random packing: (a) total length of overlaps across all edges, (b) average length of overlap on the overlapping edges, (c) the maximum overlap length, (d) the total length of gaps across all edges (e) average length of gaps on non-overlapping edges, (f) the maximum gap length on such edges.
- If all the above quantities are within 5 percent of their numerical values in the real bulk of the hypothalamus, this configuration is retained; otherwise, another random packing is proposed. The dependence of our results on the 5 percent threshold were checked (see Appendix C).

This procedure was used to generate an ensemble of 48,300 random configurations, effectively sampling the region of the phase space of configurations where the packing of subregions is comparable to the tight but somewhat elongated packing of the real hypothalamus bulk, as seen in Figure 1B.

Finally, we also investigate the network of Euclidian distances among the sub-regions of hypothalamus to find out whether the regions are organized such that the sum of distances is minimal. We define the wiring cost as:

(2) $$W=\underset{ij}{\sum }d_{ij}$$

Which assumes equal cross-sectional area for all the axonal connections. It should be noted that even though $\frac{I_{ij}}{d_{ij}}$ has the dimensions of the cross-sectional area, its asymmetric nature indicates that it does not represent the material’s cross-sectional area. To understand whether Eq. (2) is minimized in mouse hypothalamus, we follow the same procedure as in the study of Eq. (1): we use the same $10^{4}$ random packings of the 26 spheres as the starting points and use the same minimizer function of the SciPy with the same method, SLSQP, and apply the same overlap constraints.

## Results

**Figure 3:**
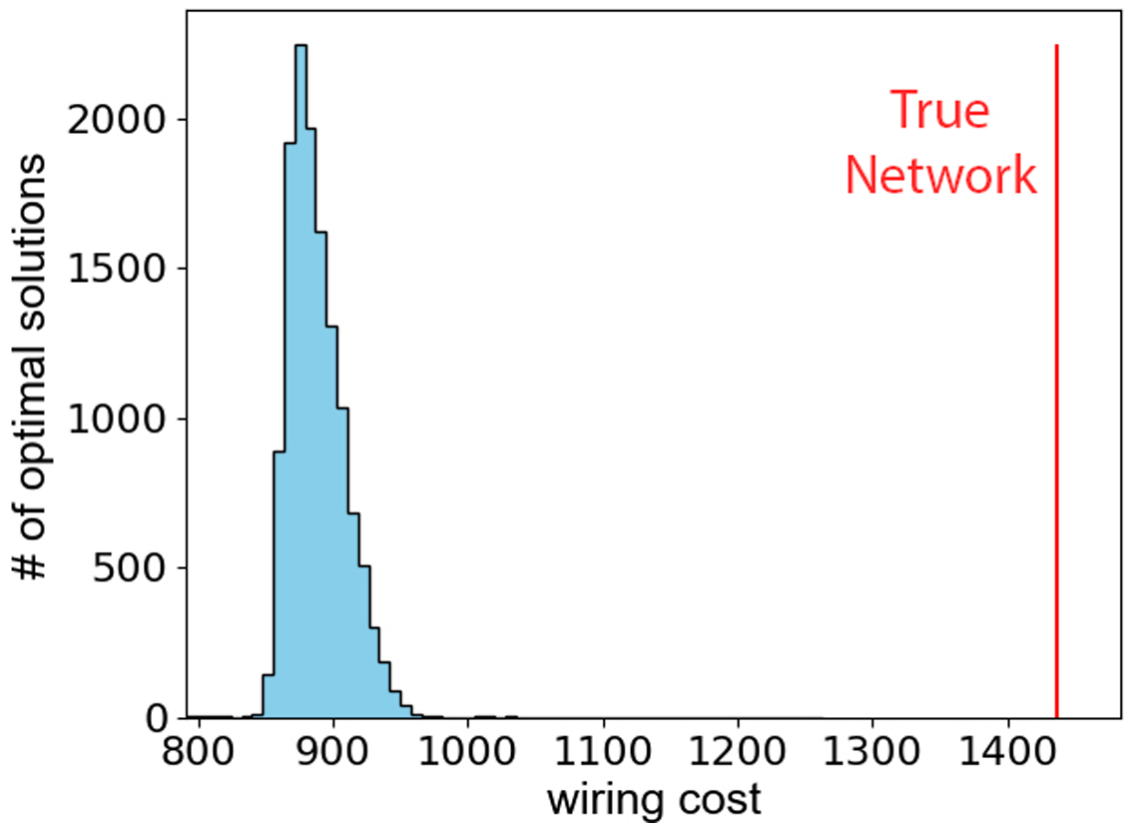
The wiring cost, defined in Eq. (2), is minimized for 10^4^ different starting points in the 26×3 dimensional phase-space. The histogram shows the optimal wiring costs. The vertical red line represents the wiring cost of true hypothalamus.

We observe that the hypothalamus as reported in the Allen Atlas does not minimize the wiring cost as defined in Eq. (2). The conclusion is reached after investigating the minimal value of the wiring cost by minimizing Eq. (2) with 10,000 different initial conditions. Figure 3 shows the resulting histogram of $10^{4}$ packings that minimize the wiring cost, and the vertical red line shows the wiring cost of the true hypothalamus. The difference between the true wiring cost and the theoretical minimal wiring cost shows that the hypothalamus is likely not organized to minimize distances between its sub-regions. Furthermore, a sub-analysis of the network properties of the hypothalamus in Appendix C confirms that network communication efficiency is not optimized in the hypothalamus, but the network does display small-world characteristics.

**Figure 4:**
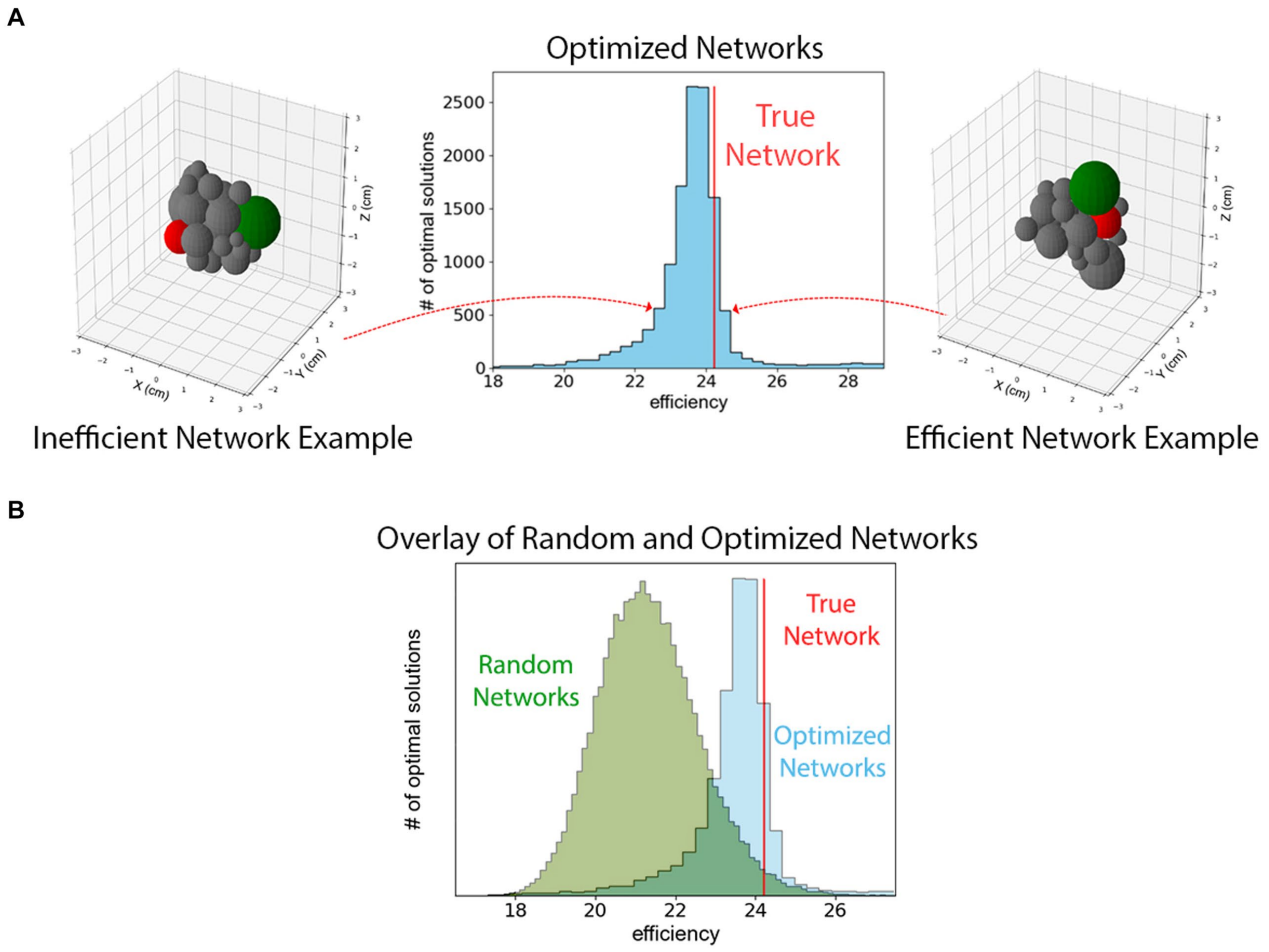
**(A)** A histogram of maximized efficiencies as defined in Eq. (1) for 10,000 random initial guesses. The vertical red line represents the efficiency of the mouse hypothalamus using the measurements derived from the Allen Brain Atlas. This figure indicates that the hypothalamus is organized such that Eq. (1) is among the highest values that are possible although it is not the unique efficient packing. The configurations on the left and right show example configurations from different low- and high-efficiency bins. Two different hypothalamus sub-regions are highlighted in green and red to show their changes in position. **(B)** The histogram of efficiencies of 48,300 randomly packed hypothalamus sub-regions superimposed upon histogram from panel **(A)**. These configurations are randomly generated and not optimized for efficiency. Comparing the two histograms shows that the true network is among the most efficient of optimized networks, which together are more efficient than an ensemble of randomly configured theoretical hypothalamic networks.

On the contrary, we observe that the spatial and axonal structure of the mouse hypothalamus is extremely efficient but not unique in terms of the definition in Eq. (1). To reach this conclusion, we used the optimizer class in SciPy library to maximize Eq. (1). Since the optimizer requires a starting point in the phase-space, we repeated the maximization process $10^{4}$ times, each with a random spatial distribution of 26 spheres as the starting points. Figure 4 shows a histogram of the optimal solutions with the red line representing the efficiency of the true hypothalamus organization. As can be seen from the figure, all $10^{4}$ trials lead to maximum efficiency values comparable to that of the true model. The figure also indicates that the efficiency measure of Eq. (1) has multiple local maxima in the 26×3 dimensional phase-space.

Additionally, we observe that the efficiency of the hypothalamus is larger than what would be expected from random packings of the regions. This observation is the result of investigating the efficiency of 48,300 randomly repacked configurations of regions in the bulk of the hypothalamus, with the conditions that (i) they have the same physical volume as the true model (ii) the neuronal connectivity and the magnitude of the projection signals, i.e., the weighted adjacency matrix $I_{ij}$ , is the same as in the true model. Furthermore, we only consider the spherical packings where overlap and gap measures between the edges are comparable to the same quantities in the true model. Figure 2B shows the histogram of the efficiencies of such random packings. The vertical line representing the efficiency of the true bulk of the hypothalamus, is at a 94th percentile on the sample of efficiencies, and about 1.6 standard deviations above the mean on the sample; suggesting that increased efficiency may be a driving factor behind the choice of configuration in the real hypothalamus.

## Discussion

Dating back to Ramón y Cajal, those studying the nervous system have argued that organizational principles include conserving material, time, and space (Ramon y Cajal, 1995; Laughlin and Sejnowski, 2003; Kaiser and Hilgetag, 2006; Budd and Kisvárday, 2012). Some works have investigated conserving connection costs, deriving principles such as wiring cost or activity-based map formation (Rubinov et al., 2015; Imam and Finlay, 2020). Others have emphasized topological properties of organization, such as topological efficiency and robustness (Achard and Bullmore, 2007; Lynall et al., 2010). Overall, however, it appears that the brain’s connectome is optimized neither to minimize connection cost nor maximize topological properties; instead, it is configured as a result of organizational tradeoffs between physical network costs and adaptive topological advantages (Bullmore and Sporns, 2012).

In particular, spatial embedding may represent an important physiological feature driving organizational principles of the brain’s connectome. By constructing a simplified, volume-based model of the hypothalamus, our organizational principle integrated both spatial packing between regions and weighted structural connectivity to derive a new metric by which to measure hypothalamic efficiency. This approach differs from previous works which focus on connection cost solely and further integrates the constraints given projection intensity as a possible measure of axonal metabolism to build a more complete model which can be seen as an extension of Bullmore and Sporns’ conclusions on the economy of brain network organization (Bullmore and Sporns, 2012).

We found that there is a significant relationship between the hypothalamic regions’ spatial orientation, axonal projection strength, and functionally efficient organization. In a model defined by simplified spherical hypothalamic structures, the hypothalamus’ organization is driven by its structural efficiency. The hypothalamus in its true configuration yielded the highest structural efficiency from our model. We observe that the hypothalamus is assembled in part due to a function of the axonal projection strength and distance between pairs of brain regions. Additionally, we observe that the wiring cost is not minimized as seen in Figure 3. However, if the distances are weighted by the magnitude of the signals that pass through them, the sum is among the lowest minimum values. Nevertheless, the hypothalamus is not in an entirely unique state that optimizes the efficiency as we have defined it as; Figure 3 shows that multiple theoretical hypothalamus configurations lead to similar or greater efficiencies than the true configuration of the hypothalamus and its sub-regions. We surmise that the reason is the existence of other competing factors than the two that we have considered. These factors might be known drivers of network organization such as network modularity (Zamani Esfahlani et al., 2021), or hub structure (van den Heuvel and Sporns, 2013), or it could be some collection of unknown factors to be discovered. Together, these findings may help drive hypothalamic development (Swanson, 1986), physiology, and pathology.

In the adult rodent brain, brain regions with similar gene expression profiles have similar connectivity profiles, and brain regions which are connected have similar expression patterns (French and Pavlidis, 2011). The spatial range of connections is likely limited suggesting that topology is biased toward neighborhoods of similarly connected regions. Thus, clustering and modularity of the brain in a spatial sense also harmonizes with a minimized wiring cost (Bullmore and Sporns, 2012). This would also explain why wiring cost is not globally minimized since these anatomical neighborhoods exist which must communicate with other modules elsewhere in the brain. Long-distance connections between hubs are costly in terms of wiring cost, but such “streets between neighborhoods” likely reduce overall energy consumption. If one applies this thinking to the organization of the entire brain, it is understandable why distinct anatomical regions, such as the hypothalamus, occur with a composition of neurons with similar connectivity and thus similar function. If one extends this thinking to the hypothalamus and its substructures, as in this investigation, then it stands to reason that the hypothalamus is organized similarly. Within the hypothalamus, itself a compartmentalized portion of the brain, sub-regions of neuronal populations with similar functions and similar connectivity are organized in topological neighborhoods with minimized wiring cost to adjacent neighborhoods. Thus, if the hypothalamus’ sub-regions are constrained by geometric volume to reduce energy costs, and then places sub-regions which communicate frequently close together, our study shows that this results in an efficient hypothalamus similar to the true rat hypothalamus (Figure 4).

While this application of large mouse connectivity datasets strives to explore novel concepts founded on prior work while remaining computationally viable in its objective, we acknowledge several limitations of our study. Much of our data relies on the Allen Brain Connectivity Atlas which in its formation implemented several quality control measures to ensure sound, yet high throughput, connectivity data. Still, the Atlas cannot guarantee that signal projections are segmented accurately with its algorithmic approach, and, among other inaccuracies, some passing fibers may be mistaken for terminal zones, biasing our data (Kuan et al., 2015). Additionally, the Atlas’ resolution is limited by its number of injection experiments. We chose only to include regions which were injection targets in our analysis because other regions are likely not mapped with significant resolution to be useful in our analysis; unfortunately, this meant sacrificing interesting regions, such as the median eminence, in our model. Our model also greatly simplifies the topological structure of the mouse brain by approximating regions as a spherical construction of their volume rather than their true geometry in order for our analysis to remain computationally feasible. This may over- or underestimate distance between projection sites, affecting our computation of efficiency. Penultimately, our analysis of the Connectivity Atlas is limited by the techniques used to gather the connectome data; specifically, although significant steps were taken by the Atlas authors to limit viral vector mapping to axons only, we cannot rule out that some data presented is based on dendritic projection data. Lastly, our study limits its analysis to the hypothalamus, and conclusions herein may not extend to the rest of the brain. Nevertheless, together these data support the hypothesis that spatial and topological efficiency contribute to the overall structure and organization of the hypothalamus.

Connectivity in the mouse brain has already been applied to animal models of human behavior through functional MRI and, in some cases, has detected alterations consistent with those detected in humans (Xu et al., 2022). Comparisons of the human and mouse connectome suggest similarities in inhibitory-to-excitatory balance and total synaptic input despite millions of years of evolutionary divergence, differing mostly in network size and interneuronal network complexity (Loomba et al., 2022). In direct studies of human disease, the connectome’s importance is highlighted by prior work which not only implicated specific cell types in autism spectrum disorder and schizophrenia, but also revealed differences in neuronal and synaptic structure spatially localized to specific cortical layers (Lynall et al., 2010; Sweet et al., 2010; Major Depressive Disorder Working Group of the Psychiatric Genomics Consortium et al., 2018; Velmeshev et al., 2019; Maynard et al., 2021). While translational research continues in this field, unwrapping network properties and enhancing our understanding of the mouse connectome lays a promising groundwork for understanding human neurological function and disease in the future.

Ultimately, neurological or psychiatric disease may be accounted for by inefficient brain organization that impacts the costliest components of processing or behavior, in terms of axonal projection strength or distance. Our findings have identified the efficiency of the hypothalamus to be an important organizer of its spatial and topological structure, and our results provide the framework for future studies to interrogate this network in the context of neurological and psychiatric disorders.

## Data availability statement

The data presented in the study are deposited in the GitHub repository hellothisisnathan/brain-efficiency, DOI: 10.5281/zenodo.12803299.

## Author contributions

NS: Conceptualization, Data curation, Formal analysis, Funding acquisition, Investigation, Methodology, Project administration, Resources, Software, Supervision, Validation, Visualization, Writing – original draft, Writing – review & editing. SA: Conceptualization, Data curation, Formal analysis, Funding acquisition, Investigation, Methodology, Project administration, Resources, Software, Supervision, Validation, Visualization, Writing – original draft, Writing – review & editing. SM: Conceptualization, Methodology, Validation, Visualization, Writing – original draft, Writing – review & editing. JK: Conceptualization, Methodology, Resources, Supervision, Validation, Writing – review & editing. JS: Conceptualization, Investigation, Resources, Supervision, Writing – review & editing. JH: Conceptualization, Investigation, Methodology, Project administration, Supervision, Visualization, Writing – original draft, Writing – review & editing. AB: Conceptualization, Investigation, Project administration, Software, Supervision, Writing – original draft, Writing – review & editing.

## Footnotes

^1^The efficiency of the subnetwork contained in this bulk of the hypothalamus is 23.5 or about 3% less than the efficiency of the entire hypothalamus network.
