# Supplementary material for: The neuroanatomical organization of the hypothalamus is driven by spatial and topological efficiency
Source: Front Syst Neurosci. 2024 Aug 5;18:1417346. doi: 10.3389/fnsys.2024.1417346 (PMC11334159; doi:10.3389/fnsys.2024.1417346)
Supplement: Supplementary file 1 [file Table_1.DOCX]

**Appendix A**

Abbreviations

| **Abbreviation** | **Allen Brain Atlas Sub-Region Name** |
| --- | --- |
| TMv | Tuberomammillary nucleus, ventral part |
| PVH | Paraventricular hypothalamic nucleus |
| PVHd | Paraventricular hypothalamic nucleus, descending division |
| ADP | Anterodorsal preoptic nucleus |
| AHN | Anterior hypothalamic nucleus |
| PVp | Periventricular hypothalamic nucleus, posterior part |
| LHA | Lateral hypothalamic area |
| LM | Lateral mammillary nucleus |
| ARH | Arcuate hypothalamic nucleus |
| LPO | Lateral preoptic area |
| AVPV | Anteroventral periventricular nucleus |
| SCH | Suprachiasmatic nucleus |
| SBPV | Subparaventricular zone |
| PSTN | Parasubthalamic nucleus |
| SO | Supraoptic nucleus |
| STN | Subthalamic nucleus |
| MM | Medial mammillary nucleus |
| MPN | Medial preoptic nucleus |
| MPO | Medial preoptic area |
| SUM | Supramammillary nucleus |
| TU | Tuberal nucleus |
| VMH | Ventromedial hypothalamic nucleus |
| ZI | Zona incerta |
| DMH | Dorsomedial nucleus of the hypothalamus |
| PH | Posterior hypothalamic nucleus |
| PMv | Ventral premammillary nucleus |

**Appendix B**

To reduce computational complexity, our model assumes the 3D space occupied by each brain region approximates to a sphere of the same volume derived from the Allen Brain Atlas. We, therefore, explore whether this is a valid assumption as it may negatively impact our predicted placements of hypothalamic sub-regions. To do so, we determine the sphericity of each brain region in its true shape and position and compare this to our approximated spherical model and computed optimized position. If there is a significant correlation between a region’s sphericity and the distance between its computed position and the true, Atlas-defined position, our spherical assumption may be invalid.

The sphericity of an object may be defined as:

$$\Psi=\frac{\pi^{1/3}\left( 6V_{p} \right)^{2/3}}{A_{p}}$$

where $V_{p}$ is the volume of the object and $A_{p}$ is its surface area. An object with a sphericity equal to 1 is a sphere and as an object becomes less spherical its sphericity decreases to a value between 1 to 0.

The Allen Brain Atlas provides the mesh representation in addition to the volume of each brain sub-region. Utilizing the mesh data, we produced a 3D reconstruction of each hypothalamic region using the Trimesh^35^ library for the Python programming language. After reconstructing the region, we were then able to obtain its surface area computationally using Trimesh. We then compared each region’s sphericity to its distance from its Atlas-defined, true position compared to our optimized hypothalamic model using linear regression. We defined this as the configuration’s error and averaged the error for each region over the top five percent (n=2415) most efficient hypothalamic configurations. The results of this analysis are shown in Fig. S1.


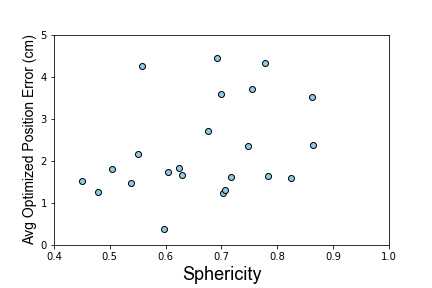


**Figure S1. A scatter plot showing the average error between each region’s optimized position and its true position** is not significantly correlated to its sphericity.

Figure S1 shows no significant correlation (y=3.11x+0.202, r=0.324, p=0.131) between a sub-region’s sphericity and its error in optimization compared to true position. Thus, we can assume that estimating hypothalamic sub-regions to be spheres is a valid model of their volume and has little effect on optimization in our algorithm.

**Appendix C**

**Network Representation:**

Graphical representations of the mouse hypothalamus connectome are presented below. Directed arrows between two regions represent non-zero projection volume from one region to the other. Figures S2, S3, and S4 represent the connections that remain upon imposing three different levels of cutoff on projection volume: removing any edges with projection volumes below 0.001, 0.02, and 0.1 respectively.


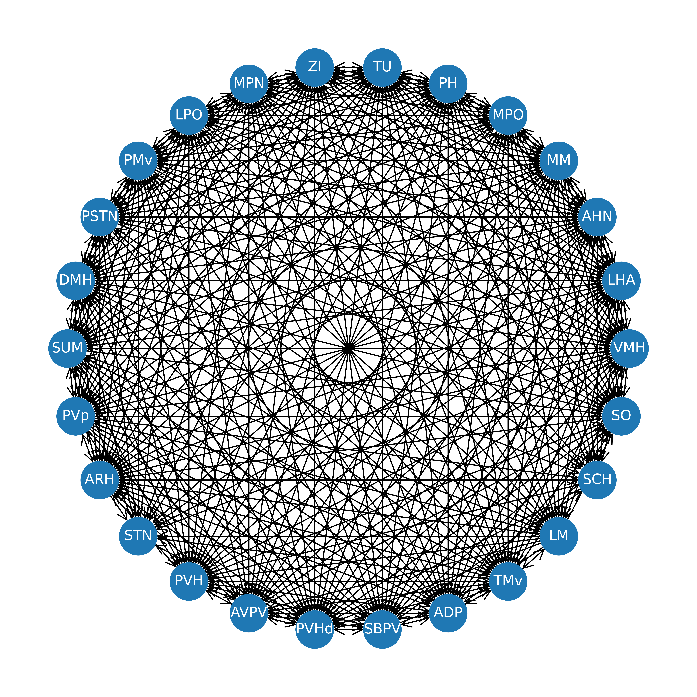


**Figure S2. An unweighted, directed graph of the mouse hypothalamus,** derived from the Allen Brain Atlas, with an axonal projection volume cutoff of 0.001.


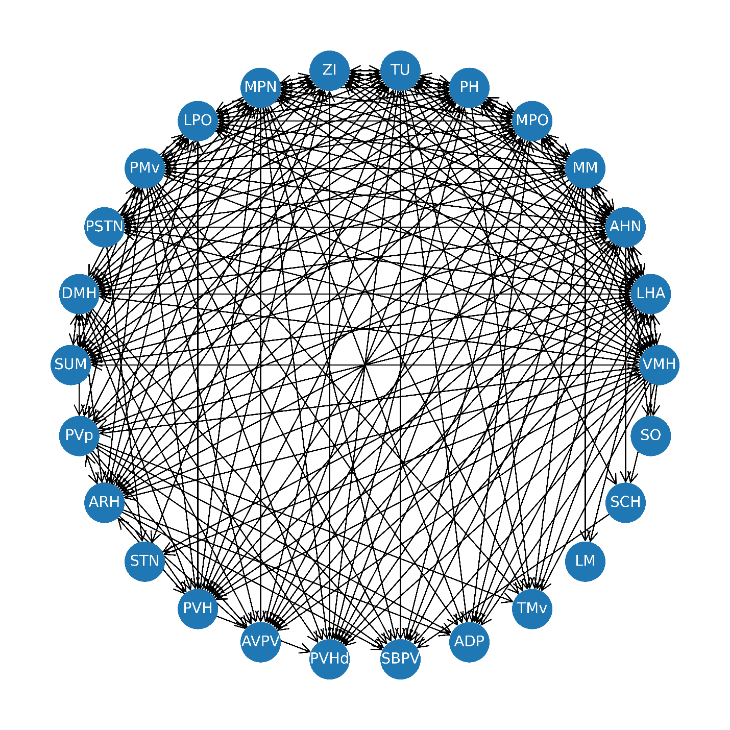


**Figure S3. An unweighted, directed graph of the mouse hypothalamus,** derived from the Allen Brain Atlas, with an axonal projection volume cutoff of 0.02.


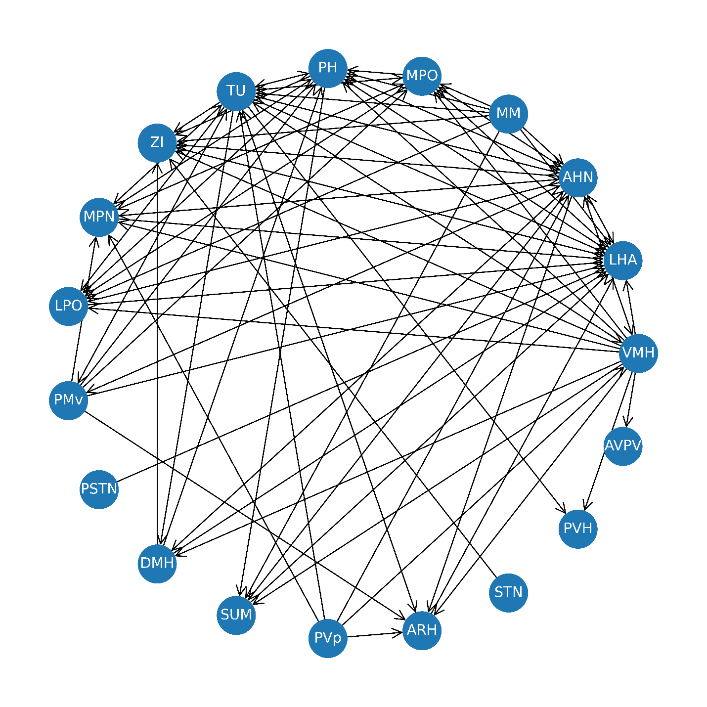


**Figure S4. An unweighted, directed graph of the mouse hypothalamus,** derived from the Allen Brain Atlas, with an axonal projection volume cutoff of 0.1.

**Betweenness-Centrality:**

Betweenness-centrality measures were computed for each region in the hypothalamus using algorithms from the software package NetworkX.^36^ In this analysis, the network is taken as a graph with directed, but unweighted edges. The edges of the graph are occupied based projection volumes that remain after imposing a cutoff. Betweenness-centrality values were evaluated for three levels of cutoff at 0.001, 0.02, and 0.1 respectively and the results are presented in Figures S5, S6, and S7.


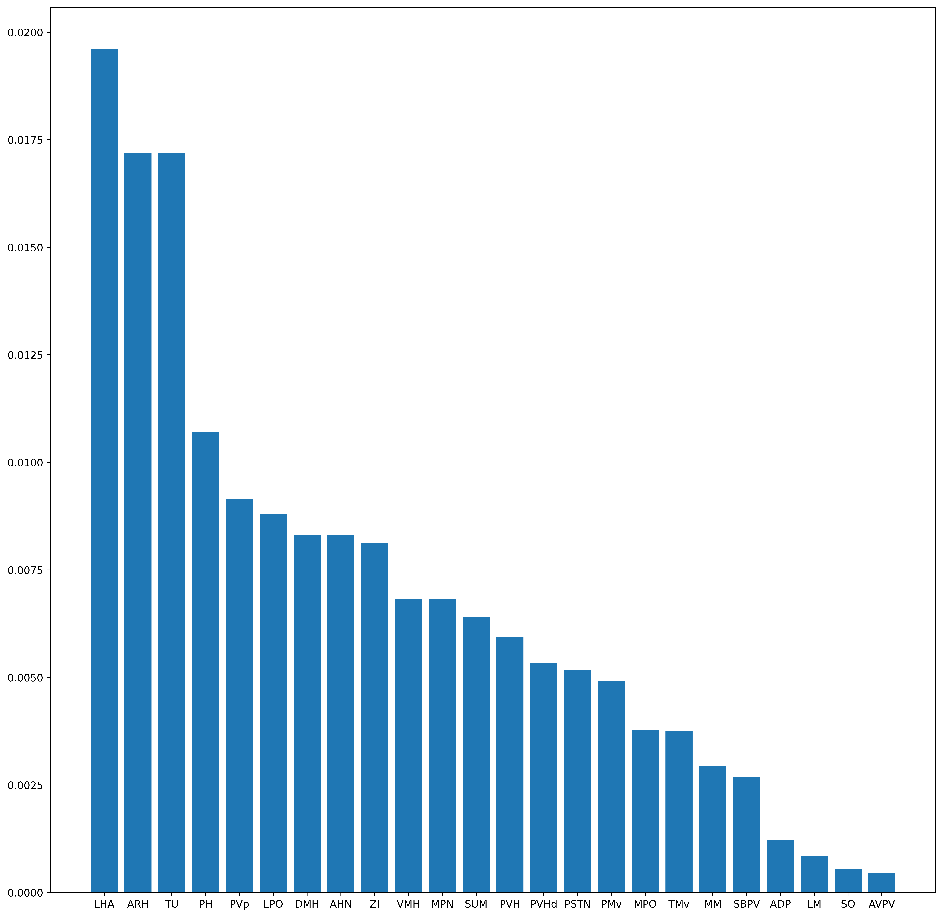


Betweenness-Centrality

**Figure S5. A bar chart of the hypothalamus’ brain regions betweenness-centrality,** derived from the graph generated by the mouse connectome, with a betweenness-centrality cutoff of 0.001.


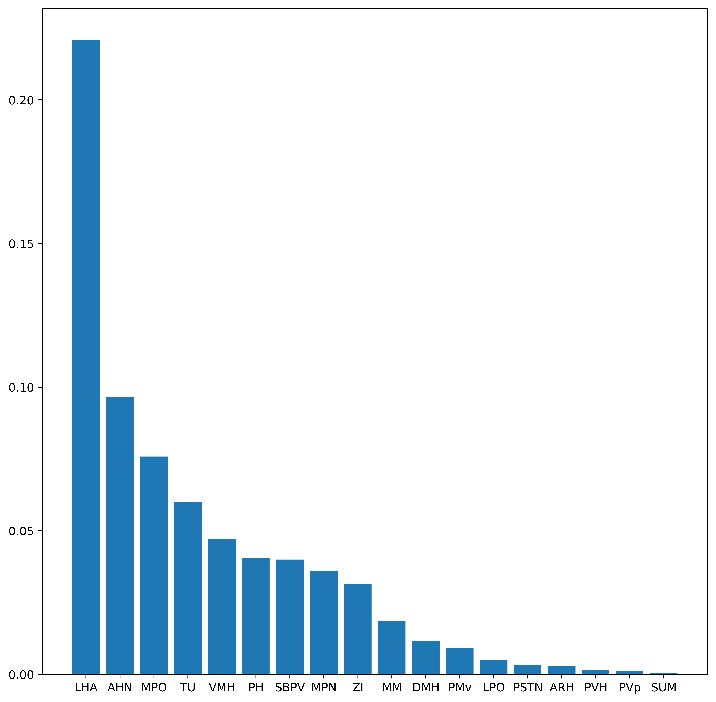


Betweenness-Centrality

**Figure S6. A bar chart of the hypothalamus’ brain regions betweenness-centrality,** derived from the graph generated by the mouse connectome, with a betweenness-centrality cutoff of 0.02.


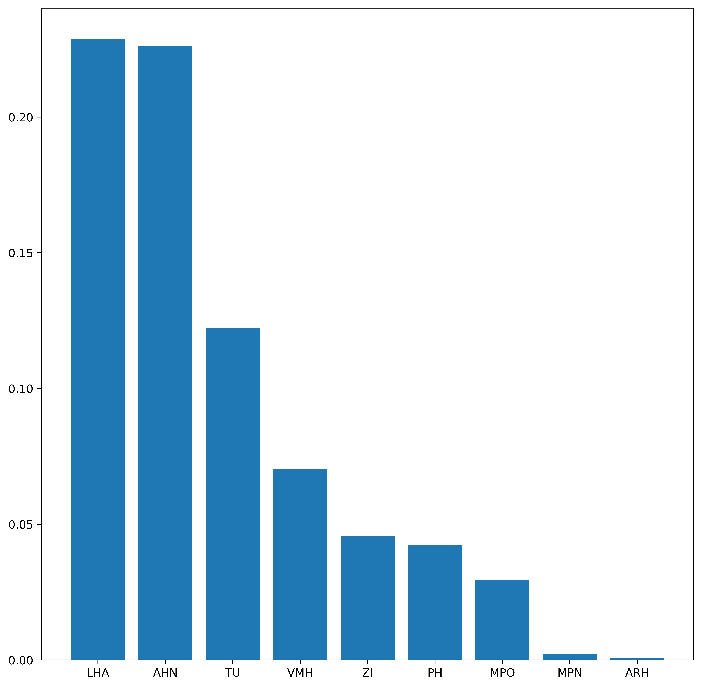


Betweenness-Centrality

**Figure S7. A bar chart of the hypothalamus’ brain regions betweenness-centrality,** derived from the graph generated by the mouse connectome, with a betweenness-centrality cutoff of 0.1.

**Small-World Coefficients:**

We use NetworkX algorithms to evaluate the small-world coefficients $\sigma$ and $\omega$ on unweighted and undirected representations of the hypothalamus connectome. These coefficients measure the small-worldedness of the network in comparison to a comparable random graph. A graph with $\sigma>1$ has small-world characteristics. $\omega$ ranges between -1 and 1; with values close to 0 implying small-world characteristics.

With cutoff levels chosen as before, $\sigma$ coefficients are evaluated to be 1.000955457482142, 1.0128133542720874, 1.0088579817808303 respectively. $\omega$ coefficients are calculated to be: -0.0010010010010008674, -0.00776472380245985, 0.0005607599947221598 respectively. Together this can be interpreted as evidence of small-world properties in the network of projection volumes in the hypothalamus.

**Local and Global Efficiencies:**

We evaluated these efficiency coefficients as defined in the NetworkX package, on the unweighted, undirected representation of the network. At the different levels of cutoff as used previously, the local efficiencies are evaluated to be 0.9783859256685343, 0.9266382239528017, 0.7296173125120493 respectively. The global efficiencies are evaluated to be 0.9692307692307692, 0.7902564102564102, 0.6929824561403517 respectively.

The sensitivity of these coefficients to the choice of cutoff order is evident.

**Number of Randomly Packed Configurations:**

Z-tests can be used to establish that the 48,300 random configurations we generated is enough to sufficiently sample the space of configurations. For example, we split the ensemble into two and run a z-test on the distribution of efficiencies over the two ensembles. With a p-value of 0.130, we are unable to reject the null hypothesis that the mean efficiencies of the two ensembles are the same.
